# Challenges Presented by Re-Emerging Sexually Transmitted Infections in HIV Positive Men who have Sex with Men: An Observational Study of Lymphogranuloma Venereum in the UK

**DOI:** 10.4172/2155-6113.1000329

**Published:** 2014-08-01

**Authors:** Minttu Rönn, Gwenda Hughes, Ian Simms, Cathy Ison, Sarah Alexander, Peter J White, Helen Ward

**Affiliations:** 1 Department of Infectious Disease Epidemiology, School of Public Health, Imperial College London, London, UK; 2 STI Section at the Centre for Infectious Disease Surveillance and Control, Public Health England, London, UK; 3 Sexually Transmitted Bacteria Reference Unit, Public Health England, London, UK; 4 MRC Centre for Outbreak Analysis and Modelling and NIHR Health Protection Research Unit in Modelling Methodology, Department of Infectious Disease Epidemiology, School of Public Health, Imperial College London, London, UK; 5 Modelling and Economics Unit, Centre for Infectious Disease Surveillance and Control, Public Health England, London, UK

**Keywords:** Lymphogranuloma venereum, HIV, Men who have sex with men, Surveillance

## Abstract

**Background:**

United Kingdom has reported the largest documented outbreak of lymphogranuloma venereum (LGV), a re-emerging sexually transmitted infection (STI) which is primarily seen in HIV-positive men who have sex with men (MSM). A diagnostic service was established in response to the outbreak linked to a voluntary LGV Enhanced Surveillance system. We examined the performance of this novel surveillance system to identify utility in tracking a re-emerging infection.

**Methods:**

We described laboratory data on samples and surveillance data from case reports for LGV from 2004-2010. We performed a cross-sectional analysis comparing clinical and behavioural characteristics of HIV-positive and HIV-negative/unknown LGV cases diagnosed in MSM using multivariable logistic regression models with generalised estimating equations to control for repeat infections.

**Results:**

LGV Surveillance data were available for 87% (1,370/1,581) of LGV cases (after de-duplication). There were 1,342 episodes in 1,281 MSM, most of whom were known to be HIV-positive (1,028/1,281, 80.2%,). HIV-positive men reported a shorter duration of symptoms (aOR 0.5; 95%CI 0.3, 0.8 for reporting more than a week compared to a week or less) in comparison to HIV-negative/unknown MSM, and were more likely to report unprotected receptive anal intercourse (aOR 2.7; 95% CI 1.3, 5.8).

**Conclusion:**

The surveillance identified the population at greater risk of infection based on higher levels of risk behaviour in HIV-positive LGV cases. However, there was diagnostic bias towards HIV-positive LGV cases who presented with a shorter duration of symptoms when compared to HIV-negative/unknown LGV cases.

## Introduction

Lymphogranulomavenereum (LGV) is a biovar of *Chlamydia trachomatis*, which causes a more invasive infection compared to non-LGV chlamydia. LGV became increasingly rare in the Western world after introduction of antimicrobials and LGV re-emergence was first noticed in the Netherlands [1] with LGV clusters appearing later in many large European and North American cities. [2]. From the early outbreaks, LGV has now established as a low level endemic STI among men who have sex with men (MSM) in large European metropolitan areas. In a case-finding exercise conducted in genitourinary medicine (GUM) clinics in London and Brighton LGV was diagnosed in 1.2% (95% CI 0.8, 1.6) of the MSM who had a sexual health screen [3]. The most comprehensive surveillance data comes from the United Kingdom (UK) and the Netherlands, with the UK having the largest documented outbreak of LGV [4-6].

The re-emergence of LGV has occurred in the context of increasing STI diagnoses in MSM both in the UK, and internationally [7,8], and other emerging STIs in HIV-positive MSM [9,10]. LGV represents an infection with severe sequel if untreated and it has managed to re-emerge and establish itself despite the control measures put in place. The emergence of LGV involved characteristics which made the public health response difficult including lack of awareness among clinicians and people at risk, a lack of simple diagnostic tests as chlamydia positive tests need subtyping to confirm LGV, prolonged treatment for LGV cases in comparison to non-LGV chlamydia, limited pre-existing surveillance and difficulties in partner notification due to high number of anonymous partners. LGV re-emergence may have been facilitated by the inconsistent clinical practice in the UK and elsewhere with case definitions and surveillance methods varying between countries [11,12]. UK responded to LGV re-emergence by establishing novel surveillance systems to cover diagnostics, and LGV Enhanced Surveillance for 2004-2010.

Maintaining and improving effective surveillance of STIs remains a priority for healthcare systems in the presence of changing epidemiological landscape: there is a growing population of people with HIV, increasing antimicrobial resistance to gonorrhoea, the re-emergence of ‘old’ STIs such as LGV and syphilis, and a rise in infections which were not traditionally considered STIs but that are able to spread through sexual contact, such as shigella and hepatitis C. The recent emergence of LGV offers a case study of how health care systems respond to and are able to control outbreaks of STIs to which little surveillance or diagnostic capacity exist prior to the emergence. This study will address and evaluate the utility of the enhanced surveillance system for LGV in the UK, by firstly looking at the type of samples tested for LGV giving a context to general testing patterns, and then examining the LGV Enhanced Surveillance system.

There is a strong association between HIV and LGV [13] and in this study we compared the characteristics of LGV cases with and without diagnosed HIV to assess differences in their behavioural and clinical profile.

## Material and Methods

The case definition of LGV requires confirming the presence of LGV serovar (L1-L3) in a sample positive for *Chlamydia trachomatis* [14]. Since 2004 the Sexually Transmitted Bacteria Reference Unit (STBRU), Public Health England (PHE; formerly known as the Health Protection Agency) has provided a diagnostic service for LGV. Tests were available for genital and rectal specimens from MSM attending genitourinary medicine (GUM) clinics who were diagnosed with *C. trachomatis* and had symptoms suggestive of LGV or were contacts of an LGV-positive patient [15,16]. The diagnostic service was initially for the whole of the UK and since August 2006, Scottish specimens have been referred to the Scottish Bacterial Sexually Transmitted Infections Reference Laboratory. Samples arriving to STBRU for LGV testing are first re-screened for *C. trachomatis* (by real-time PCR and independent primers [14]). A small proportion (5-10%) of the samples degrade during transit, and some test negative for *C. trachomatis* due to variation in sensitivity of different testing methods (Sarah Alexander, STBRU, personal communication).

A voluntary enhanced clinical surveillance system for LGV (LGVES) was introduced in 2004 as part of an outbreak response in order to gain a better understanding of risk factors. The system was discontinued at the end of 2010 and replaced with routine surveillance through the GUM clinic activity dataset (GUMCAD; www.hpa.org.uk/gumcad). LGVES was paper-based with a form consisting of 24 main questions and associated sub-questions [17] which was completed by the clinician retrospectively (based on clinical notes and/or following consultation with the patient) after the patient had been confirmed to have LGV. It included information on demographic (gender, sexuality, age, ethnicity, city of GUM clinic), clinical (date of clinic presentation, reason(s) for attending, site of infection, date of symptoms onset, types of symptoms, treatment, concurrent STIs at LGV presentation) and behavioural (probable country of acquisition, locations/sources of meeting new sex partners, number of sex partners and recent sexual practices) factors. LVGES forms were collected and maintained by the STI Section at the Centre for Infectious Disease Surveillance and Control in Colindale, PHE. For the analysis HIV status was determined based on items 14-18 on the surveillance form and episodes were determined as HIV-positive or HIV-negative/unknown if the form did not clearly indicate the patient being HIV-positive. We compared cases presenting before 2010 to those presenting during 2010 to explore a sudden increase in case-numbers observed during 2010 and potential impact this might have had on the case profile. Duration of symptoms was categorised as above or below 7 days which was the mode of the distribution.

We built explanatory statistical models (as opposed to predictive models) to determine which variables were associated with HIV status at LGV diagnosis. Missing data were coded as an unknown category to evaluate the effect of differential reporting. Two preliminary multivariable models were constructed: one for clinical factors and another for behavioural factors. From these, a final multivariable model of variables that were considered to be of interest for the HIV-LGV association, or to be potential confounders for this association according to *a priori* hypotheses and objectives, was produced. For secondary selection criteria p-value 0.2 was used as a cut-off point for statistical associations of potential interest.

We also had information on LGV re-infection at clinic level. Where two episodes in the same individual occurred within three months, the second was excluded as a possible duplicate notification, treatment failure or rapid re-infection from untreated partner [18]. Episodes belonging to the same individual are more likely to be similar than episodes belonging to different individuals, and therefore analysis was performed at individual-level allowing clustering for repeat infections. We performed the statistical analyses using a logit model and generalised estimating equations (GEE) and robust standard errors. GEE, is a population-average model, which takes into consideration correlation within clusters, but assumes no between-cluster correlation [19]. An exchangeable correlation structure was chosen as large differences in the correlation structure were not expected [20]. We used Wilcoxon rank sum test to estimate differences in medians in delay of LGV Enhanced Surveillance form filling [21]. We performed the analyses using Stata/SE 11.2.

## Results

### Study population

A total of 10,566 *C. trachomatis* positive samples, where the sex of the patient was recorded, were tested for LGV at STBRU between 2004-2010; the majority were from rectal swabs: 91.1% for men (9,138/10,035) and 84.9% for women (451/531). In men, 15.5% (1,417/9,138) of rectal swabs were found to have LGV. Urethral swab, urine and throat swab LGV positivity was 7.1% (11/156), 2.9% (4/136) and 2.7% (4/151), respectively. Only 0.8% of samples from women (4/531) tested positive for LGV, all from rectal swabs. LGVES forms were available for 87% (1,370/1,581) of cases after de-duplication [6].

Of these 1,370 confirmed LGV episodes, we excluded 28 for this study (3 from women, 2 in those of unknown sex, 5 in heterosexual men, 10 in men with unknown sexuality and 8 which occurred within 3 months of a previous episode in the same individual), leaving 1342 episodes in MSM for the analysis. Of these, 1,087 (81%) were in 1,028 HIV-positive and 255 in 254 HIV-negative/unknown men; all but one of the recorded LGV re-infections occurred in the HIV-positive group. The median time between the patient presenting to the clinic and LGVES form being filled was 98 days (range 3-1,574 days; median of 96 days for the HIV-positive and 101 for the HIV-unknown; p-value for the difference 0.776).

### Clinical presentation among LGV cases

The association between clinical correlates and HIV status among the cases is presented in Table 1. HIV-positive men were more likely to report any systemic symptoms and the association was independent of other clinical factors (adjusted odds ratio [aOR]1.6, 95% CI 1.0, 2.4), but they were less likely to report symptoms for more than one week prior to clinic presentation compared to HIV-negative/unknown men (aOR 0.5, 95% CI 0.3, 0,8). HIV-positive men were less likely to have information missing on concurrent STIs (aOR 0.5, 95% CI 0.3, 0.9), or to be referred from another clinician to the STI/HIV clinic (aOR 0.3, 95% CI 0.1, 0.8) but referral was a rare reason for attending the clinic in general. There was little information on genital LGV as few patients reported genital site of infection or genital symptoms; HIV-positive men were less likely to report genital LGV in the univariable analysis but the association ceased to be statistically significant in the multivariable analysis.

### Risk behaviour among LGV cases

The association between behavioural variables and HIV status among the LGV cases is shown in Table 2. Being HIV-positive and reporting unprotected receptive anal intercourse (RAI) had the strongest significant association amongst the behavioural variables (aOR 3.1, 95% CI 1.5, 6.1); although HIV-positive men were also almost three times more likely to have information on RAI missing this was not statistically significant. HIV-positive men were more likely to report both unprotected insertive and receptive fisting though this was not significant and there were few events in the HIV-negative/unknown group (aOR 3.7, 95% CI 1.0, 14.1), and there was a poor response on the variable in half of the episodes. HIV-positive men were less likely to report bisexual orientation, and this association remained significant in the multivariable model (aOR 0.2, 95% CI 0.1, 0.7), however only 23 LGV episodes were reported in this category. Unlike in the clinical multivariable model, presenting with LGV during 2010 remained associated with HIV status in the behavioural multivariable model (aOR 1.5, 95% CI 1.0, 2.3).

### Final multivariable model

The purpose of the concluding multivariable model was to explore the potential joint effects of clinical and behavioural factors by including the most relevant variables together (presented in Table 3). In the final model, HIV-positive men were less likely to report more than a week of symptoms prior to clinic attendance (aOR 0.5, 95% CI 0.3, 0.8) and to have information on concurrent STIs missing (aOR 0.5, 95% CI 0.3, 1.0). Unprotected RAI had the strongest significant association with being HIV-positive (aOR 2.7, 95% CI 1.3, 5.8) whilst reporting bisexuality retained its negative association (aOR 0.2, 95% CI 0.1, 0.6).

## Discussion

The public health response to the re-emergence of LGV involved the establishment of new surveillance systems, specialised diagnostic methods, and raising awareness among clinicians and MSM. Centralising the diagnostic service at STBRU meant that it was possible to monitor the coverage of the LGVES system. The LGVES was able to describe the key population at risk for LGV, namely a small subpopulation of HIV-positive MSM reporting diverse and higher risk sexual practices. We found that HIV-positive cases reported higher levels of risk behaviour than HIV-negative/unknown LGV cases which would support this idea. However, our analyses also demonstrated that HIV-negative/unknown MSM have a longer duration of symptoms before presentation to the clinic compared to HIV-positive men suggesting that they were tested for LGV less frequently than HIV-positive men; they also had less complete reporting of concurrent STI diagnoses further suggesting there may be systematic differences in STI screening of MSM depending on their HIV status.

The type of analysis performed here can offer a retrospective evaluation of the surveillance system and of LGV re-emergence. Due to the delay in LGV Enhanced Surveillance form filling this type of surveillance is less suited for a real-time outbreak analysis; however the laboratory surveillance for LGV can detect changes in case numbers quickly assuming the coverage of LGV testing is sufficient. The retrospective nature of data collection is also subject to recall bias and as the information is based on clinician’s notes or interviews with the patient the data are subjected to desirability bias. The time delay in clinic presentation to form filling was added to all multivariable models, and we included unrecorded responses as a separate category for the explanatory variables. This was done to measure and adjust for the nature of the data collection. This can reduce but it is unlikely to fully eliminate the biases in the data. The analysis was further limited by the definition of HIV status we had available. The LGVES form did not specify whether the HIV-negative men were tested for HIV and there is potential for misclassification of the outcome. This is likely to dilute the associations seen between the explanatory variables and the outcome.

Initial control efforts for LGV were unable to curb the re-emergence and it appears to have become endemic within this sub-population. This may in part be due to the limited detection of asymptomatic cases. The first case-finding exercise in the UK identified predominantly symptomatic LGV cases [3,4], but more asymptomatic cases have been found since [22] and in the Netherlands almost half of the cases have been reported as asymptomatic [5,23,24]. This occurs in the context of inconsistent clinical practice regarding screening MSM for rectal chlamydia, based on a national survey in GUM clinics, which found that cases are likely to be missed until/unless they develop severe symptoms [25]. This can limit the detection of cases and the utility of a surveillance system if it is biased towards symptomatic cases.

Similar problems are seen with syphilis, hepatitis C and HIV among MSM where, despite availability of testing and treatment, the control efforts are not sufficient to bring down population level incidence [26]. This would imply that the targeting or the frequency of testing is not high enough to reduce onward transmission. Changes in the population structure may also contribute to the emergence of infectious diseases if they increase the number of susceptible and exposed individuals [27]. For LGV and other STIs the increasing prevalence of HIV diagnosed men, and their changing sexual behaviours in the presence of a life-long infection, is likely to contribute to STI re-emergence. This has been postulated as a factor for syphilis in Germany [9].

Control of infectious diseases requires effective surveillance, which consists of systematic collection and analysis of data of occurrence and transmission of the infection leading to dissemination of results and action based on the evidence [28].However, collecting unbiased data through national surveillance can be challenging [29] emphasising the need for periodic validation of the data collected using additional sources of data on prevalence and epidemiology of the infection. Lessons learnt from LGV surveillance can help us with future re-emerging STIs. For novel outbreaks, there is a need to gain information of the epidemiology to target testing and control efforts accordingly whilst for ongoing epidemics routine data can highlight issues which can be examined more thoroughly with focused enhanced surveillance. Regardless of the surveillance data collected, it should be continuously reviewed for suitability and validity and the accumulating evidence should feedback to the system for it to adapt to changes in the epidemic curve and risk profile of cases.

For LGV – which remains a rare endemic STI in the UK – this can be achieved through regular case-finding exercises. This also allows a more reliable estimation of the true burden and distribution of infection, and it is more likely to capture changes in epidemiological profile of cases. Although comprehensive, enhanced LGV surveillance did not measure recreational drug use and related problems now considered to be important risk factors in certain STI epidemics in MSM [7,30]. These and other unforeseen aspects of risk behaviours can be discovered by qualitative research, which has proven particularly useful for shigella outbreaks in MSM [30]. For rare infections, understanding the specific context for transmission becomes especially important for appropriate control measures and prevention messages.

As the epidemic matures the surveillance should do the same and move on from outbreak investigation (aiming to identify all cases with detailed information collected) into an ongoing surveillance (surveillance system which is feasible given the costs associated whilst maintaining good data quality). Further exploration of the best methods for such surveillance is important if we are to address the rising challenges of emerging infections and antibiotic resistance.

## Figures and Tables

**Table 1:** Clinical variables and their association with HIV status in the LGV Enhanced Surveillance data.

	HIV-positive	HIVnegative/unknown	Univariable logistic regression (GEE)		Multivariable logistic regression (GEE)^a^	
	n=1087	n=255	OR (95% CI)	p-value	(OR (95% CI)	p-value
**Presentation year**						
Before 2010	731 (67.7%)	196 (77.2%)	1.0		1.0	
During 2010	349 (32.3%)	58 (22.8%)	1.6 (1.2, 2.2)	0.003	1.0 (0.7, 1.5)	0.989
**Episode number**						
1st	1023 (94.1%)	254 (99.6%)	Not included		Not included	
2nd	58 (5.3%)	1 (0.4%)				
3rd	6 (0.6)	0 (0.0%)				
**Clinic in London**						
No	316 (29.1%)	100 (39.2%)	1.0		1.00	
Yes	771 (70.9%)	155 (60.8%)	1.5 (1.2, 2.1)	0.003	1.4 (0.9, 2.0)	0.099
**Duration of symptoms**						
Week or less	377 (34.7%)	65 (25.5%)	1.0		1.00	
More than a week	500 (46.0%)	141 (55.3%)	0.6 (0.5, 0.8)	0.002	0.5 (0.3, 0.8)	0.002
Unknown	210 (19.3%)	49 (19.2%)	0.7 (0.5, 1.1)	0.146	0.7 (0.4, 1.3)	0.304
**Reasons for attending**						
**Symptoms**						
No	139 (12.9%)	31 (12.2%)	1.0		Not included	
Yes	925 (85.1%)	218 (85.5%)	0.9 (0.6, 1.4)	0.783		
Unknown	23 (2.1%)	6 (2.4%)	0.8 (0.3, 2.1)	0.710		
**Contact tracing**						
No	980 (90.2%)	229 (89.8%)	1.0		Not included	
Yes	84 (7.7%)	20 (7.8%)	1.0 (0.6, 1.6)	0.927		
Unknown	23 (2.1%)	6 (2.4%)	0.9 (0.4, 2.1)	0.769		
**Routine STI screen**						
No	988 (90. 9%)	232 (91.0%)	1.0		Not included	
Yes	76 (7.0%)	17 (6.7%)	1.1 (0.6, 1.8)	0.849		
Unknown	23 (2.1%)	6 (2.4%)	0.9 (0.4, 2.1)	0.779		
**Referral**						
No	1,033 (95.0%)	233 (91.4%)	1.0		1.0	
Yes	31 (2.6%)	16 (6.3%)	0.4 (0.2, 0.8)	0.011	0.3 (0.1, 0.8)	0.015
Unknown	23 (2.1%)	6 (2.4%)	0.8 (0.4, 2.0)	0.713	0.6 (0.2, 1.6)	0.292
**Symptomsreported**						
None	60 (5.5%)	11 (4.3%)	1.0		1..0	
Only Genital	41 (3.8%)	24 (9.4%)	0.3 (0.1, 0.7)	0.005	0.5 (0.2, 1.2)	0.107
Only Rectal	758 (69.7%)	147 ( 57.7%)	1.0 (0.5, 1.8)	0.880	1.2 (0.5, 2.8)	0.600
Both	172 (15.8%)	54 (21.2%)	0.6 (0.3, 1.2)	0.140	0.7 (0.3, 1.7)	0.403
Unknown	56 (5.2%)	19 (7.5%)	0.6 (0.3, 1.3)	0.167	0.9 (0.3, 2.4)	0.810
**Site of infection**						
Rectal	454 (41.8%)	63 (29.3%)	1.0		1.0	
Genital	10 (0.9%)	5 (2.3%)	0.3 (0.1. 0.9)	0.032	0.6 (0.2, 2.1)	0.377
Both or other	8 (0.7%)	3 (1.2%)	0.4 (0.1, 1.5)	0.182	0.6 (0.1, 2.4)	0.428
Unknown	614 (56.5%)	144 (97.0%)	0.5 (0.4, 0.7)	<0.001	0.6 (0.4, 0.9)	0.015
**Systemic symptom**						
No	750 (69.0%)	186 (72.9%)	1.0		1.0	
Yes	292 (26.9%)	57 (22.4%)	1.3 (0.9, 1.7)	0.150	1.6 (1.0, 2.4)	0.034
Unknown	45 (4.1%)	12 (4.7%)	0.9 (0.5, 1.8)	0.833	1.5 (0.6, 3.3)	0.360
**Other STI** ^b^						
No	664 (61.1%)	143 (56.1%)	1.0		1.0	
Yes	315 (29.0%)	72 (28.2%)	1.0 (0.7, 1.3)	0.718	1.2 (0.8, 1.8)	0.404
Unknown	108 (9.9%)	40 (15.7%)	0.59 (0.4, 0.9)	0.010	0.5 (0.3, 0.9)	0.013
**Hepatitis C (PCR)**						
No	410 (37.7%)	57 (22.4%)	1.0		Not included	
Yes	138 (12.7%)	3 (1.2%)	5.9 (2.0, 17.5)	0.001		
Unknown	539 (49.6%)	195 (76.5%)	0.4 (0.3, 0.5)	<0.001		

a Multivariable model adjusted also for form delay (days between presentation to the clinic and filling in the surveillance form)

b No other STIs versus syphilis, non-specific urethritis, warts, herpes, hepatitis B and some less common STIs mentioned in the text field.

**Table 2:** Behavioural variables and their association with HIV status in the LGV Enhanced Surveillance data.

	HIV-positive	HIVnegative/unknown	Univariable logistic regression (GEE)		Multivariable logistic regression (GEE)^a^	
	(n=1087)	(n=255)	OR (95% CI)	P-value	OR (95% CI)	P-value
**Presentation year**						
Before 2010	731 (67.7%)	196 (77.2%)	1.0		1.0	
During 2010	349 (32.3%)	58 (22.8%)	1.6 (1.2, 2.2)	0.003	1.5 (1.0, 2.3)	0.034
**Age**						
mean (sd)	38.6 (8.1)	37.0 (9.9)	1.0 (1.0, 1.0)	0.023	1.0 (1.0, 1.0)	0.072
**Ethnicity**						
White	953 (87.7%)	229 (89.8%)	1.0		Not included	
Black	51 (4.7%)	9 (3.5%)	1.3 (0.7, 2.8)	0.426		
Asian	30 (2.8%)	7 (2.8%)	1.0 (0.4, 2.3)	0.969		
Other	36 (3.3%)	7 (2.8%)	1.2 (0.5, 2.7)	0.650		
Unknown	17 (1.6%)	3 (1.2%)	1.3 (0.4, 4.4)	0.625		
**Sexuality**						
Homosexual	1,076 (99.0%)	243 (95.3%)	1.0		1.0	
Bisexual	11 (1.0%)	12 (4.7%)	0.2 (0.1, 0.5)	<0.001	0.2 (0.1, 0.7)	0.008
**Acquisition country**						
UK	828 (76.2%)	198 (77.7%)	1.0		1.0	
Abroad	81 (7.5%)	16 (6.3%)	1.2 (0.7, 2.1)	0.476	1.1 (0.5, 2.3)	0.812
Either	47 (4.3%)	13 (5.1%)	0.9 (0.5, 1.6)	0.651	0.7 (0.3, 1.7)	0.497
Unknown	131 (12.1%)	28 (11.0%)	1.1 (0.7, 1.7)	0.604	0.8 (0.4, 1.7)	0.634
**Met sex partners in sex-on-premises venues** ^b^						
None reported	277 (25.5%)	63 (24.7%)	1.0		1.0	
Met partners in the locations	263 (24.2%)	50 (19.6%)	1.2 (0.8, 1.8)	0.371	0.8 (0.4, 1.3)	0.302
Unknown	547 (50.3%)	142 (55.7%)	0.9 (0.6, 1.2)	0.449	0.7 (0.5, 1.1)	0.182
**Number of contacts**						
median (range)	3 (0-201)	3 (0-213)	1.0 (1.0, 1.0)	0.354	1.0 (1.0, 1.0)	0.134
**Receptive anal intercourse**						
None reported	51 (4.7%)	24 (9.4%)	1.0		1.0	
Protected/or unknown^c^	161 (14.8%)	79 (31.0%)	0.9 (0.5, 1.6)	0.840	1.4 (0.6, 3.0)	0.440
Unprotected	791 (72.8%)	127 (49.8%)	2.9 (1.7, 4.8)	<0.001	3.1 (1.5, 6.1)	0.002
Unknown	84 (7.7%)	25 (9.8%)	1.6 (0.8, 3.0)	0.180	2.8 (1.0, 8.3)	0.057
**Insertive anal intercourse**						
None reported	100 (9.2%)	25 (9.8%)	1.0		1.0	
Protected/or unknown^c^	154 (14.2%)	65 (25.5%)	0.6 (0.4, 1.0)	0.056	0.8 (0.4, 1.6)	0.556
Unprotected	615 (56.6%)	106 (41.6%)	1.5 (0.9, 2.3)	0.117	1.1 (0.6, 2.1)	0.803
Unknown	218 (20.1%)	59 23.1	0.9 (0.6, 1.6)	0.811	1.1 (0.5, 2.6)	0.736
**Any oral sex**						
None reported	72 (6.6%)	28 (11.0%)	1.0		1.0	
Reported some	23 (2.1%)	6 (2.4%)	1.5 (0.6, 3.9)	0.439	1.8 (0.5, 6.3)	0.382
Reported one unprotected	30 (2.8%)	10 (3.9%)	1.2 (0.5, 2.7)	0.728	1.1 (0.3, 3.0)	0.976
Reported both unprotected	812 (74.7%)	165 (64.7%)	1.9 (1.2, 3.0)	0.008	1.6 (0.7, 3.5)	0.289
Some or all unknown	150 (13.8%)	46 (18.0%)	1.3 (0.7, 2.2)	0.414	0.8 (0.3, 2.1)	0.689
**Any fisting**						
No fisting reported	419 (38.6%)	114 (44.7%)	1.0		1.0	
Some fisting reported	52 (4.8%)	9 (3.5%)	1.6 (0.8, 3.2)	0.224	2.1 (0.7, 6.3)	0.170
Both reported, unprotected	71 (6.5%)	6 (2.4%)	3.1 (1.4, 6.9)	0.007	3.7 (1.0, 14.1)	0.056
Some unknown	545 (50.1%)	126 (49.4%)	1.2 (0.9, 1.6)	0.245	1.1 (0.7, 1.7)	0.655
**Sharing sex toys**						
None reported	412 (37.9%)	108 (42.4%)	1.0		Not included^d^	
Any reported	77 (7.1%)	17 (6.7%	1.2 (0.7, 2.0)	0.581		
Unknown	598 (55.0%)	130 (51.0%)	1.2 (0.9, 1.6)	0.193		
**Vaginal intercourse**						
None reported	798 (73.4%)	201 (78.8%)	1.0		Not included	
Any reported	4 (0.4%)	2 (0.8%)	0.5 (0.1, 2.6)	0.415		
Unknown	285 (26.2%)	52 (20.4%)	1.4 (1.0, 1.9)	0.051		

a Multivariable model adjusted also for form delay (days between presentation to the clinic and filling in the surveillance form).

b No new sex partners in these locations versus backroom, sauna, cruising ground and sex party.

c Reported the type of sex without information on protection, or reported the type of sex was protected.

d Sharing sex toys not included in the multivariate model as it was strongly correlated (covariance >0.8) with fisting variable.

**Table 3:** Final multivariable model.

Multivariable logistic regression (GEE) ^a^		
	aOR (95% CI)	P-value
**Presentation year**		
Before 2010	1.0	
During 2010	1.1 (0.7, 1.8)	0.736
**Seen in a clinic in London**		
No	1.0	
Yes	1.1 (0.7, 1.7)	0.648
**Duration of symptoms**		
Week or less	1.0	
More than a week	0.5 (0.3, 0.8)	0.002
Unknown	0.7 (0.4, 1.4)	0.369
**Referral**		
No	1.0	
Yes	0.4 (0.2, 1.1)	0.073
Unknown	0.4 (0.2, 1.2)	0.107
**Location of symptoms reported**		
None	1.0	
Only Genital	0.4 (0.1, 1.2)	0.118
Only Rectal	1.4 (0.6, 3.4)	0.441
Both	0.8 (0.3, 2.0)	0.567
Unknown	0.9 (0.3, 2.8)	0.879
**Site of infection**		
Rectal	1.0	
Genital	1.2 (0.2, 5.5)	0.847
Both or other (throat, n=1)	0.5 (0.1, 2.0)	0.303
Unknown	0.6 (0.4, 1.0)	0.053
**Any systemic symptom**		
No	1.0	
Yes	1.6 (1.0, 2.6)	0.057
Unknown	2.0 (0.8, 5.1)	0.157
**Any other STI**		
No	1.0	
Yes	1.1 (0.7, 1.7)	0.601
Unknown	0.5 (0.3, 1.0)	0.037
**Age**	1.0 (1.0, 1.1)	0.099
**Sexuality**		
Homosexual	1.0	
Bisexual	0.2 (0.1, 0.6)	0.006
**Number of contacts**	1.0 (1.0, 1.0)	0.370
**Receptive anal intercourse**		
None reported	1.0	
Reported protected or unknown	1.4 (0.6, 3.3)	0.428
Unprotected	2.7 (1.3, 5.8)	0.010
Unknown	2.5 (0.8, 7.6)	0.109
**Insertive anal intercourse**		
None reported	1.0	
Reported protected or unknown	0.8 (0.4, 1.6)	0.473
Unprotected	1.2 (0.6, 2.2)	0.668
Unknown	1.3 (0.5, 2.7)	0.641
**Any oral sex**		
None reported	1.0	
Reported some	1.9 (0.5, 7.0)	0.336
Reported one unprotected	1.2 (0.4, 4.0)	0.730
Reported both unprotected	1.9 (0.9, 4.2)	0.108
Some or all unknown	1.0 (0.4, 2.5)	0.992
**Any fisting**		
No fisting reported	1.0	
Some fisting reported	1.9 (0.6, 6.3)	0.297
Both reported, unprotected	3.5 (0.8, 15.5)	0.093
Some unknown	0.9 (0.6, 1.4)	0.777

a Multivariable model adjusted also for form delay (days between presentation to the clinic and filling in the surveillance form).

## Abbreviations

CI: Confidence Interval
GEE: Generalised Estimating Equations
GUM: Genitourinary Medicine
HIV: Human Immunodeficiency Virus
LGV: Lymphoranuloma venereum
LGVES: LGV Enhanced Surveillance
MSM: Men Who Have Sex with Men
OR: Odds Ratio
PHE: Public Health England
STBRU: Sexually Transmitted Bacteria Reference Unit
STI: Sexually Transmitted Infection
